# Surgical Cases

**Published:** 1876-06

**Authors:** Charles C. F. Gay

**Affiliations:** Surgeon to the Buffalo General Hospital


ART. III. — *Surgical Cases.* By CHARLES C. F. GAY, M. D., Surgeon to the Buffalo General Hospital.

INGUINAL HERNIA, WITH UNUSUAL STRANGULATION—OPERATION. — On November 1st, 1874, I was invited by Dr. Hauenstein to see with him Mr.——, a German, aged 45 years, who was suffering with what appeared to be strangulated ingunial Hernia.

The following history of the case was obtained: The Hernia was 21 years old. Truss had never been worn. The bowel sometimes was returned into the abdominal cavity, but usually was allowed to remain within the scrotum, as no inconvenience resulted therefrom until now. Three or four days ago it became troublesome. The whole of the scrotum became swollen, inflamed, and consequently painful. No passage could be obtained from the bowels; there was occasional vomiting. Timely and judicious attempts by taxis, to reduce the hernia had been made in vain by his medical attendant.

The scrotum was held up by suspensory bandage, and a lotion of acetate of lead had been used, which had the effect to allay the inflammation. A cathartic had been administered, which neither operated nor aggravated the symptoms. Vomiting was not stercoraceous; there was no peritonieal tenderness nor inflammation; pulse 108 per minute.

The protruding bowel extended to the fundus of the scrotum, and was two inches or two inches and a half in diameter.

Passing my thumb and forefinger up along its course, I found the tumour terminates before reaching the external abdominal ring, which is evidence that the stricture does not exist at the ring. I next determined to ascertain the nature of the contents of the tumour, whether fluid or solid, for this purpose made use of the aspirator and drew off only a few drops of fluid.

An operation was then decided upon and made at once with the assistance of Drs. Hauenstein and Bartow.

Chloroform was given at first, and its effect maintained afterwards by ether. Upon cutting down to the ring I found that I could easily pass my finger into the abdominal cavity. There was certainly no stricture there.

Following the downward course of the intestine I came upon the stricture which was in the sac, and three inches below the external ring.

The stricture was very firm and was divided by cutting from above downwards. The sac was then laid open and its walls found to be a quarter of an inch in thickness. Adhesions extended down into the scrotum; were broken up, and the bowel returned into the abdominal cavity. Although the operation would have been completed after division of the stricture, it was thought advisable to return the extruded bowel rather than allow it to remain where, indeed, it had been immured in safety for many years. Its appearance was not at all inviting. There was evidence of approaching gangrene, the color was bad and the intestine was cold and fully impacted with fecal matter, which gentle pressure failed to unload. In 48 hours the patient succumbed—autopsy not allowed.

Femoral Aneurism so Closely Simulating Femoral Hernia as to Render Diagnosis Difficult.—On the eleventh of March last, (1876) I visited at Corfu, Genesee county, Mrs. D., aged 67 years, who was reported to have strangulated femoral hernia.

9

The following history I obtained from her medical attendant:

Four years ago a small tumour was first observed just below the flexure of the thigh toward the inner aspect. When the patient lies down the tumor disappears to reappear again when standing up. It was never until the present time, painful or troublesome. A truss had never been worn.

Three days before my visit the tumor had suddenly enlarged to the size of a pullet's egg, and had become painful; scarcely any nourishment could be retained; a cathartic, repeated, with repeated enemas, had failed to open the bowels, and this was the condition of the patient when I saw her. On examination of the tumour, I was wholly unable to say positively that it was or was not hernia. The tumour occupied the position below Poupart's ligament where femoral protrusion occurs, yet rather lower down upon the thigh than is usual for hernia. It was over the femoral artery, yet there was no pulsation in the tumour either direct or transmitted. Taxis had been employed but the tumour was now so tender to the touch that any attempt at manipulation gave great distress.

Ether was now administered, when, assisted by Drs. Congar, and Bartow, I cut down upon the tumor, passed a ligature around the superficial femoral artery—without tying it—in order to guard against accident from hemorrhage, then dissected around the tumour when it was found that the artery ran into it, and was continuous with it below. The ligature was then tightened, another one placed upon the artery below the tumour, the sac was freely incised and the aneurisual clot turned out. The wound was brought together by interrupted silver sutures and the case progressed to a favorable termination.

The wound however did not close by primary union, but opened on removal of sutures and healed by granualation.

The ligatures came away on the twenty-first day.

The method employed in this case it may be observed, was that of Antyllus, modified.

Dislocation of the Left Femur into the Foramen Thyroideum.—Reduction by the Reid Method of Manipulation.— By request of Dr. Diehl, I visited, along with himself and Dr.

Johnson, on August 4th, 1875, Mr. B——, aged 51 years, a man full six feet in height with good muscular developement. During a scuffle his feet slipped laterally, separating his limbs widely apart, in which attitude he fell across a chair and down upon the floor, from which he found himself unable to rise without assistance. I saw him soon after the accident; extending the two legs, scarcely any difference was perceptible in their length. The luxated limb was perhaps a trifle longer than its fellow. The toe was everted; nobility of the limb was not impaired; by rotating the limb, the head of the femur was felt in the obturator foramen.

Chloroform was administered, and after two failures at reduction, the third trial was successful and was accomplished in the following manner:

The leg was flexed upon the thigh and the thigh flexed upon the body, and carried over the opposite limb. In this position the head of the femur could be felt to move in the foramen, but when rotating the limb outward the head could not be felt, but whenever the reverse maneuvre was made, namely: rotation *inward*, the head was felt to move outward and backward toward the acetabulum, when, by quickly extending the limb the head of the bone was made to pass into its socket.

But a very few minutes were occupied in this last effort at reduction.

The limb was secured in position by appropriate bandaging. Much discoloration of the integument existed over and around the hip for a few days, but the patient made a good recovery.

Should diagnosis be difficult in a luxation of the femur into the foramen, it may be made sure by feeling for the head of the bone through the rectum.

Reduction was made in less than thirty minutes, and was accomplished by the joint efforts of Drs. Conrad Diehl, Chas. H. Johnson and myself.

